# Measuring indirect transmission-reducing effects in tuberculosis vaccine efficacy trials: why and how?

**DOI:** 10.1016/S2666-5247(23)00112-X

**Published:** 2023-08

**Authors:** Kristin N Nelson, Gavin Churchyard, Frank Cobelens, Willem A Hanekom, Philip C Hill, Benjamin Lopman, Vidya Mave, Molebogeng X Rangaka, Johan Vekemans, Richard G White, Emily B Wong, Leonardo Martinez, Alberto L García-Basteiro

**Affiliations:** a Department of Epidemiology, Rollins School of Public Health, Atlanta, GA, USA; b Aurum Institute, Parktown, South Africa; c Department of Global Health and Amsterdam Institute for Global Health and Development, Amsterdam University Medical Centers, University of Amsterdam, Amsterdam, Netherlands; d Africa Health Research Institute, KwaZulu Natal, South Africa; e Centre for International Health, University of Otago, Dunedin, New Zealand; f Johns Hopkins Center for Infectious Diseases in India, Pune, India; g Institute for Global Health and MRC Clinical Trials Unit, University College London, London, UK; h V4R, Brussels, Belgium; i Tuberculosis Modelling Group, Department of Infectious Disease Epidemiology, London School of Hygiene & Tropical Medicine, London, UK; j Division of Infectious Diseases, Department of Medicine, Heersink School of Medicine, University of Alabama Birmingham, Birmingham, AL, USA; k Department of Epidemiology, School of Public Health, Boston University, Boston, MA, USA; l Centro de Investigação em Saude de Manhiça (CISM), Maputo, Mozambique; m ISGlobal, Hospital Clínic–Universitat de Barcelona, Barcelona, Spain; n Centro de Investigación Biomédica en Red de Enfermedades Infecciosas (CIBERINFEC), Barcelona, Spain

## Abstract

Tuberculosis is the leading bacterial cause of death globally. In 2021, 10·6 million people developed symptomatic tuberculosis and 1·6 million died. Seven promising vaccine candidates that aim to prevent tuberculosis disease in adolescents and adults are currently in late-stage clinical trials. Conventional phase 3 trials provide information on the direct protection conferred against infection or disease in vaccinated individuals, but they tell us little about possible indirect (ie, transmission-reducing) effects that afford protection to unvaccinated individuals. As a result, proposed phase 3 trial designs will not provide key information about the overall effect of introducing a vaccine programme. Information on the potential for indirect effects can be crucial for policy makers deciding whether and how to introduce tuberculosis vaccines into immunisation programmes. We describe the rationale for measuring indirect effects, in addition to direct effects, of tuberculosis vaccine candidates in pivotal trials and lay out several options for incorporating their measurement into phase 3 trial designs.

## Introduction

Tuberculosis is one of the leading infectious causes of death, leading to 1·6 million deaths in 2021.^1^ After persistent declines in incidence for nearly two decades, the estimated number of new diagnoses of tuberculosis globally increased from 10·1 million in 2020 to 10·6 million in 2021.^1^ If current trends continue, we will fail to reach the main tuberculosis incidence and mortality reduction targets set by the WHO's End TB strategy.^2^ New tools, including new vaccines against tuberculosis, are essential to ensure that hard-won gains in tuberculosis prevention can be recovered and that global targets can be reached. Seven promising tuberculosis vaccine candidates for adolescents and adults (ie, aged >10 years) are currently in late-stage clinical trials that, if successful, would lead to vaccine approval and licensure.^3^ However, the standard designs of such trials collect little to no information on the ability of vaccines to prevent tuberculosis transmission, which is crucial for understanding how introducing a vaccine programme will affect disease incidence. In this Personal View, we provide several potential approaches to measuring indirect effects that should be considered for pivotal, or phase 3, efficacy trials of tuberculosis vaccines still in the planning stages.

Several of these promising vaccine candidates for adolescents and adults are currently in pivotal efficacy (phase 2b and 3) trials.^4^ M72-AS01_E_ (Gates Medical Research Institute, Seattle, WA, USA; GSK Biologicals, Rixensart, Belgium), a recombinant protein subunit vaccine that is given in two doses one month apart, was shown in a phase 2b trial to provide 49·7% (90% CI 12·1–71·2) protection against pulmonary tuberculosis in QuantiFERON-positive (Qiagen, Hilden, Germany) adults aged 18–65 years after three years in Kenya, South Africa, and Zambia.^5^ A large, multicountry, phase 3 trial for the M72-AS01_E_ vaccine candidate is expected to begin in 2023. VPM1002 (Serum Institute of India, Pune, India), a recombinant BCG vaccine, is currently in phase 2/3 trials (NCT03152903) to test efficacy against the recurrence of tuberculosis in adults aged 18–65 years in India and Bangladesh and in a separate phase 3 trial (CTRI/2019/01/017026) to assess the prevention of disease in household contacts older than 6 years in India. GamTBVac (Gamaleya Federal Research Center for Epidemiology and Micorbiology, Moscow, Russia), a protein subunit vaccine given in two doses two months apart, is being tested in a phase 3 trial (NCT04975737) to prevent the development of pulmonary tuberculosis among BCG-vaccinated, tuberculosis-uninfected adults aged 18–45 years in Russia. MTBVAC (Biofabri, Montevedra, Spain), an attenuated live vaccine, is currently being tested in phase 2 trials (NCT02933281) in adults with and without *Mycobacterium tuberculosis* infection in South Africa, with plans to evaluate the prevention of disease in people with HIV.^6^, ^3^ Although vaccines are also under development for younger age groups, quantifying transmission-reducing effects of adult vaccines is particularly important considering that adult-type pulmonary tuberculosis is more readily transmitted from person to person than is tuberculosis that develops in young children.

As currently designed, pivotal trials are likely to underestimate the population-level effect of introducing new tuberculosis vaccines. The primary aim of phase 3 trials is, rightly, to establish the individual benefit of vaccination, which provides the basis for licensure decisions and regulatory approval. To achieve this goal, trials are designed to evaluate whether vaccines effectively prevent disease, most commonly with primary endpoints of bacteriologically confirmed tuberculosis. However, vaccines might also have indirect effects on unvaccinated individuals. Specifically, indirect effects can occur because the vaccine: (1) prevents infection in the first place and so reduces the overall number of people who can spread *M tuberculosis*, or (2) reduces the infectiousness of those who do become infected (figure), or both.^7^, ^8^, ^9^, ^10^ The type and magnitude of indirect effects of a vaccine have important implications for the overall effect of a vaccine programme. Although the primary objective of phase 3 trials should remain the evaluation of direct effects, expanding trial designs to also measure indirect effects could provide a more comprehensive understanding of the population-level effect of new tuberculosis vaccines.

**Figure:**
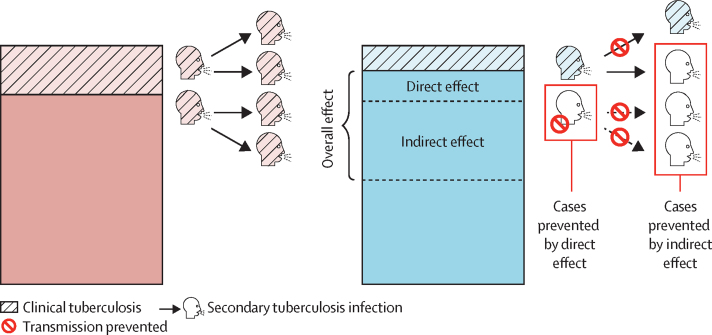
Indirect, transmission-reducing effects of tuberculosis vaccines In current designs of phase 3 vaccine trials, the direct effect of the vaccine is calculated by comparing the proportion of unvaccinated (pink) and vaccinated (blue) groups who develop clinical tuberculosis (dashed areas). Individuals who develop tuberculosis (dashed) could cause secondary tuberculosis infections (arrows), which could be prevented if the vaccine has indirect effects. A vaccine could reduce infectiousness and onward transmission in vaccinated individuals who develop tuberculosis, or prevent secondary transmission that would have otherwise occurred (dotted arrows) in the absence of a vaccine programme.

### Why measure indirect effects in trials of tuberculosis vaccines?

The effect of a vaccine programme is the sum of the direct effects of vaccination, or the biological protection conferred by the vaccine among those who are vaccinated, and the indirect effects, which afford protection to both vaccinated and unvaccinated individuals through reductions in community transmission. The indirect effects of a vaccine are often evaluated in post-licensure vaccine effectiveness studies, which commonly include cluster randomised trials, case-control studies, and household transmission studies.^11^, ^12^, ^13^, ^14^ For the BCG vaccine, at least one study has evaluated its indirect effects by comparing rates of tuberculosis in individuals who were ineligible for vaccination in areas subject to mass vaccination policies and areas that were not.^15^ However, evaluating vaccine effects is more difficult after a vaccine is proven to be efficacious, when randomising the receipt of vaccination is no longer ethical. Although non-randomised (ie, observational) study designs can be powerful, important differences between vaccinated and unvaccinated groups relating to the reasons individuals choose to be vaccinated can lead to biased conclusions. Moreover, relegating studies of indirect effects to the post-licensure period risks discarding a vaccine candidate that shows a modest direct effect but large indirect effects. For example, a 50% direct effect to individuals with *M tuberculosis* infection in preventing tuberculosis might translate into greater than 50% reductions in infection or disease at the population level (ie, direct plus indirect effects).^5^, ^16^ Although post-licensure studies of indirect effects will still be important and phase 3 trials should remain primarily focused on evaluating direct efficacy to support licensure decisions, the pivotal trial stage represents a window of opportunity to also evaluate the indirect effect of a vaccine to support initial policy recommendations that must be made before introducing the vaccine.^17^ There are at least three reasons why measuring and considering indirect effects at the pre-licensure stage would be useful.

First, estimates of indirect effects might be important for national health ministries tasked with making decisions about whether to introduce a tuberculosis vaccine programme. Although introducing a tuberculosis vaccine programme could be clearly cost-effective for some countries that have a very high tuberculosis incidence, for others the benefit might appear marginal. Some middle-income countries that receive little or no in-kind support for vaccine procurement and introduction will need to carefully balance the health effects of a tuberculosis vaccine programme with the associated costs. For countries where the cost-effectiveness of introducing a new tuberculosis vaccine is less clear, the decision to implement a vaccine programme could hinge on the magnitude of indirect effects promised by a vaccine. For example, mRNA-based SARS-CoV-2 vaccines have shown similar efficacy against severe disease, but have variable efficacy in reducing viral loads and preventing transmission.^18^, ^19^ Similar data on indirect effects might be particularly useful considering the multitude of promising tuberculosis vaccine candidates in phase 2/3 trials. Consider the possibility that two tuberculosis vaccines are approved and available for use, both with similar direct efficacy, but one reduces infectiousness whereas the other has no or little effect. The value proposition of the first vaccine might be more favourable than the second. This information would be key for policy makers and other stakeholders to have when making decisions about adoption of a tuberculosis vaccine.

Second, understanding the expected magnitude of indirect effects could support decisions about which populations to prioritise for vaccination. To evaluate the relative merits of different prioritisation strategies—by age, risk group, or otherwise—knowing the extent to which vaccinating one group might also protect others is important. If indirect effects are strong, focusing vaccination efforts on a small group with high tuberculosis incidence that could be expected to attain high coverage might be more effective at reducing tuberculosis incidence overall than vaccinating the general population. Modelling studies have shown that tuberculosis vaccination efforts targeted towards specific groups who have high tuberculosis incidence or in geographical hotspots could be more impactful than community-wide vaccination; this benefit might be even greater if a vaccine has a strong infectiousness-reducing effect, for example.^20^, ^21^

Third, information on indirect effects is essential to properly weigh the benefit of a vaccine programme against any risks that might emerge soon after introduction of the vaccine. There have been several such instances in the past: the Rotashield vaccine (Wyeth Laboratories, Marietta, PA, USA), which was introduced to the US immunisation programme in 1998, was found to cause intussusception shortly after it was licensed.^22^ More recently, administration of mRNA SARS-CoV-2 vaccines was paused in 2021 to assess a possible vaccine-linked risk of myocarditis.^23^ In both cases, public health officials were tasked with weighing the benefits of vaccination against emerging information on possible risks to formulate a policy response. Although safety concerns should be paramount, and even the promise of broad public health benefits might not outweigh an individual-level risk posed by vaccination, public health officials should be able to consider the full spectrum of benefits from vaccination, including those from indirect effects. Introducing a vaccine with a poor understanding of these benefits could hinder these high-stakes calculations.

### Approaches for measuring indirect effects of tuberculosis vaccines

We propose three approaches to assess potential indirect effects in pivotal trials (panel). First, trials could measure the extent to which the vaccine reduces infectiousness among vaccinated and unvaccinated people who develop tuberculosis. This approach is commonly implemented in trials of vaccines against viral illness, such as SARS-CoV-2, for which viral load can be readily quantified with molecular tests and is considered to be correlated with the risk of onward transmission.^24^ Although there is not an equally reliable marker of infectiousness for tuberculosis, information (eg, smear status), the extent of lung cavitation on a chest radiograph, GeneXpert (Cepheid, Sunnyvale, CA, USA) semiquantitative results,^25^, ^26^, ^27^, ^28^ and newer methods (eg, face-mask or cough aerosol sampling, which measure *M tuberculosis* in exhaled breath) might approximate expelled bacterial load and suggest differences in transmissibility of infection.^29^, ^30^ Although the precision of results from this approach might be limited by having only a small number of participants reach trial endpoints, implementing within currently proposed trial designs would be relatively straightforward.

Panel **Measures, advantages, and disadvantages of considering indirect effects of tuberculosis vaccines in late-stage clinical trials**

This panel descibes key knowledge gaps and considerations for expanding tuberculosis vaccine trials to assess indirect effects. Priority is based on the feasibility with current tools, current regulatory requirements, and cost.

**Approach 1: measure individual-level markers of infectiousness among trial participants reaching endpoints**

*Advantages*

• Less resource-intensive than other approaches

*Disadvantages*

• Infectiousness measures might be poor markers of transmission risk
• Infectiousness measure captured at one timepoint might not capture the full effect on transmission (ie, does not account for possible shorter durations of illness)
• Limited power to detect differences if few instances of tuberculosis accrue in the trial

*Priority*

• High for well-established infectiousness measures (ie, smear status and cavitation on chest x-ray)
• Medium for experimental infectiousness measures (ie, face mask or cough aerosol sampling)

**Approach 2: integrating household transmission studies in trials**

*Advantages*

• Gold standard approach for measuring transmission

*Disadvantages*

• Resource-intensive approach
• Does not capture community transmission, which might account for most transmission in settings with high tuberculosis incidence
• Little generalisability outside of household setting (efficacy against transmission may appear low given high-intensity exposure in household)
• Little power to detect differences if few instances of tuberculosis accrue in trial

*Priority*

• Medium

**Approach 3: cluster randomised trials**

*Advantages*

• Characterise full spectrum of indirect effects

*Disadvantages*

• Difficult to implement and may not generate evidence for vaccine licensure

*Priority*

• Low

Second, trials could incorporate substudies that directly measure transmission without the need to extrapolate infectiousness from bacteriological tests. Integrating household transmission studies within current trial designs, as has been done in clinical trials of pertussis^31^ and SARS-CoV-2 vaccines,^19^ would allow for comparison of transmission risk to people in close contact with vaccinated and unvaccinated participants who develop tuberculosis. A household transmission study could be conducted among the subset of participants who develop clinical tuberculosis. In this case, a diagnosis of clinical tuberculosis would trigger tuberculin skin test (TST) or interferon gamma-release assay (IGRA) testing for children in the household of the trial participant, with TST or IGRA positivity suggesting recent exposure to tuberculosis and indicating probable household transmission. Of note, trial protocols would also need to establish procedures for providing household members with tuberculosis preventive therapy. Alternatively, a random subset of trial participants could be selected whose household members would participate in active surveillance and routine TST or IGRA testing over the course of the trial to capture any conversions. Although the first approach would capture differences in transmission between vaccinated and unvaccinated participants who meet the clinical tuberculosis endpoint definition, the second would also capture transmission from participants with subclinical tuberculosis that do not present with symptoms that would prompt evaluation for trial endpoints. Careful evaluation of tuberculosis incidence rates and age-specific tuberculosis prevalence in a trial site would indicate whether either or both types of studies are feasible given the sample sizes that would be required to show differences between vaccinated and unvaccinated groups. Although household transmission studies might be limited in power and would not account for the large amount of transmission that occurs in community settings, they are worth serious consideration because they offer the most direct measurement of the extent to which the vaccine might reduce risk of transmission in vaccinated individuals.

Third, to evaluate the potential indirect effects of a tuberculosis vaccine, trial endpoint definitions could also be broadened to include other infection states besides clinical tuberculosis from which transmission can occur. According to an analysis of tuberculosis prevalence surveys in Africa and Asia, approximately 50% of patients (range 35–80%) with bacteriologically confirmed tuberculosis are negative on a symptom screen.^32^ Combined with increasing evidence that many individuals who do not report tuberculosis symptoms have high bacillary loads that suggest infectiousness, it is not surprising that modelling studies have indicated subclinical tuberculosis, or tuberculosis detectable by bacteriological or radiological tests but without clinical symptoms, could be responsible for a large proportion of *M tuberculosis* transmission.^33^, ^34^, ^35^ Given the potential role in transmission, incorporating a secondary or exploratory endpoint of subclinical tuberculosis could facilitate the measurement of indirect effects in phase 3 trials while keeping trials focused on evaluating the vaccine's ability to prevent clinical tuberculosis. This approach is comparable to the design of vaccine trials against other respiratory infections, including SARS-CoV-2, which include routine testing of participants for infection regardless of symptoms.^36^ In contrast with asymptomatic SARS-CoV-2 infection, detection of subclinical tuberculosis would prompt the initiation of treatment and reduce the likelihood of observing the primary trial endpoint of clinical tuberculosis. For this reason, an evaluation of subclinical tuberculosis once at the end of the trial might be preferred to routine evaluation. In either case, including subclinical tuberculosis as an exploratory endpoint would allow the expansion of the approaches laid out above to include participants with subclinical tuberculosis: individual-level infectiousness measures could be collected among trial participants that develop subclinical tuberculosis (panel; approach 1) and household transmission studies could be conducted among those reaching endpoints of subclinical tuberculosis (panel; approach 2). Of note, the addition of secondary or exploratory endpoints would not affect the required sample size or duration of the trial, which is based on the primary endpoint.

Finally, cluster randomised trials, in which entire communities are randomised to receive the vaccine or placebo, in principle offer the opportunity to measure both direct and indirect effects. Cluster trial designs have been used to evaluate the effectiveness of typhoid vaccines but have not, to our knowledge, been used in earlier stages of vaccine development, and are not currently considered acceptable to support licensure of a vaccine.^37^ Moreover, the design and implementation of cluster trials can be complex; they require very large sample sizes and represent the most substantial departure from current trial designs.^14^, ^38^

### Knowledge gaps

We have described several approaches that would allow assessment of the indirect, transmission-reducing effects of tuberculosis vaccine candidates in phase 3 trials. However, there are key knowledge gaps that, if filled, would facilitate such studies and further support the basis for the modifications that we propose to current trial designs.

First, developing assays that can form the basis of validated individual-level measures of infectiousness is crucial to properly compare the risk of vaccinated and unvaccinated individuals spreading tuberculosis. New methods offer the ability to measure infectiousness with efficient, non-invasive, and relatively low-cost assays, regardless of the presence of symptoms. For example, face-mask sampling techniques have shown considerable promise. Initial studies have shown that mask sampling is able to detect subclinical tuberculosis, has higher sensitivity to detect pulmonary tuberculosis than chest X-rays or sputum smear testing, and is correlated with onward transmission.^29^, ^39^, ^40^ Although these early results are encouraging, few studies have been performed, data have been collected in few settings, and sample sizes have been small.^41^ Cough aerosol sampling could also measure an individual's likelihood of transmitting tuberculosis; although this approach is not easily implemented in routine clinical settings, it could prove feasible in the context of clinical trials.^30^, ^42^ In both cases, there is still uncertainty about the strength of the link between detectable bacteria from a face-mask or aerosol sample and an individual's ability to transmit tuberculosis infection, because bacteria that are captured with these techniques might be phenotypically different from those that can produce infection. Validation studies showing that readouts from these assays are reliable markers of transmission risk would provide a strong rationale for their use in trials. Although conventional measures of infectiousness (eg, smear status and cavitation) could be immediately implemented in trials, other assays could be included but considered exploratory in nature until validation studies are complete.

Second, the evidence base for the risk of transmission from people with subclinical tuberculosis is poor. To provide additional support for the premise that measuring subclinical tuberculosis would provide information on transmission risk, there is a need for additional evidence of transmission from people with subclinical tuberculosis. At present, most of this evidence is indirect or has been inferred from modelling studies.^33^, ^34^, ^43^, ^44^, ^45^, ^46^ Additionally, there remains little consensus on the definition of subclinical tuberculosis; the defining features of this condition should be agreed upon previous to consideration as a secondary or exploratory endpoint. However, including subclinical tuberculosis as a secondary or exploratory endpoint could be considered even in the absence of the above mentioned studies.

Finally, additional work is needed to establish the feasibility of implementing the approaches we have proposed. Ongoing studies in preparation for phase 3 tuberculosis vaccine trials are focused on measuring clinical tuberculosis incidence in possible trial sites; these studies could also collect data on household transmission to determine the power and feasibility of household transmission studies and, if subclinical tuberculosis were to be considered as a secondary or exploratory endpoint, measure incidence and prevalence rates of subclinical tuberculosis. Simulation studies that use data on local tuberculosis epidemiology could compare the relative feasibility, sample size, and cost of different trial designs on the basis of local epidemiological context.

## Conclusion

Over a century has passed since a new vaccine against tuberculosis was introduced. Hopes for a new tuberculosis vaccine rest largely on several late-stage vaccine candidates that are currently poised to enter late-stage trials. Optimising trial designs to provide rich information on both the direct and possible indirect effects of vaccine candidates will provide a clear picture of their potential population-level effect. Broadening the type of data that are collected in pivotal efficacy trials can bridge the gap between vaccine development and implementation, ensuring that policy makers have the necessary evidence to make informed decisions about introducing new tuberculosis vaccines into immunisation programmes globally. As new information on the indirect effects of new tuberculosis vaccines emerges, it should be incorporated into mathematical models to improve estimates of overall health and economic effect, which are essential for global and national decision making.

## Declaration of interests

We declare no competing interests.
